# Economic optimization of full-sib test group size and genotyping effort in a breeding program for Atlantic salmon

**DOI:** 10.1186/s12711-019-0491-5

**Published:** 2019-09-03

**Authors:** Kasper Janssen, Helmut W. Saatkamp, Mario P. L. Calus, Hans Komen

**Affiliations:** 1 Wageningen University & Research, Animal Breeding and Genomics, P.O. Box 338, 6708 PB Wageningen, The Netherlands; 2 Wageningen University & Research, Business Economics, P.O. Box 8130, 6706 KN Wageningen, The Netherlands

## Abstract

**Background:**

Breeding companies may want to maximize the rate of genetic gain from their breeding program within a limited budget. In salmon breeding programs, full-sibs of selection candidates are subjected to performance tests for traits that cannot be recorded on selection candidates. While marginal gains in the aggregate genotype from phenotyping and genotyping more full-sibs per candidate decrease, costs increase linearly, which suggests that there is an optimum in the allocation of the budget among these activities. Here, we studied how allocation of the fixed budget to numbers of phenotyped and genotyped test individuals in performance tests can be optimized.

**Methods:**

Gain in the aggregate genotype was a function of the numbers of full-sibs of selection candidates that were (1) phenotyped in a challenge test for sea lice resistance (2) phenotyped in a slaughter test (3) genotyped in the challenge test, and (4) genotyped in the slaughter test. Each of these activities was subject to budget constraints. Using a grid search, we optimized allocation of the budget among activities to maximize gain in the aggregate genotype. We performed sensitivity analyses on the maximum gain in the aggregate genotype and on the relative allocation of the budget among activities at the optimum.

**Results:**

Maximum gain in the aggregate genotype was €386/ton per generation. The response surface for gain in the aggregate genotype was rather flat around the optimum, but it curved strongly near the extremes. Maximum gain was sensitive to the size of the budget and the relative emphasis on breeding goal traits, but less sensitive to the accuracy of genomic prediction and costs of phenotyping and genotyping. The relative allocation of budget among activities at the optimum was sensitive to costs of phenotyping and genotyping and the relative emphasis on breeding goal traits, but was less sensitive to the accuracy of genomic prediction and the size of the budget.

**Conclusions:**

There is an optimum allocation of budget to the numbers of full-sibs of selection candidates that are phenotyped and genotyped in performance tests that maximizes gain in the aggregate genotype. Although potential gains from optimizing group sizes and genotyping effort may be small, they come at no extra cost.

## Background

When the genetic level of a breeding stock from a breeding company is superior to that of its competitors, the breeding company may want to sell its products at a premium price or to increase its market share [1, 2]. Since genetic gain is cumulative, even small differences in the rate of genetic gain between breeding companies become significant over time. In Atlantic salmon breeding, the sale price of eggs does not vary much between breeding companies because either the differences in the genetic level of eggs are small or these differences are not reflected in the sale price of eggs. In contrast to the sale price of eggs, market shares vary a lot between salmon breeding companies [3]. The turnover of a breeding company increases with its market share. If a breeding program has a fixed cost, a larger turnover will generate higher profits. Assuming that the market share of a breeding company is related, at least partly, to the genetic level of its products, there is a clear incentive to maximize the rate of genetic gain from the breeding program with regard to the limited annual budget that is available.

Salmon breeding programs often use nested mating designs to create both full- and half-sib groups. Since in salmon, family sizes are large, a portion of the members of each family is used as selection candidates, while the remaining individuals are subjected to dedicated performance tests for traits that cannot be recorded on selection candidates, such as disease resistance and slaughter traits. These large family sizes allow for within-family genomic selection, with the use of pedigree information to predict family means and genomic information to exploit within-family variation, i.e. to estimate the Mendelian sampling terms [4]. In pedigree-based selection, accuracy increases at a declining rate with the number of test individuals that are phenotyped, such that marginal gains in the aggregate genotype decrease. Similarly, with genomic selection, accuracy increases at a declining rate with the number of test individuals that are genotyped [4, 5], such that marginal gains in the aggregate genotype decrease. In contrast, the cost of a performance test increases (more or less) linearly with the number of test individuals that are phenotyped and the number of test individuals that are genotyped. When the annual budget of a breeding company that is available for its breeding program is limited, the budget allocated to phenotyping and genotyping individuals in one performance test is at the expense of the budget for an alternative performance test. In other words, such a limited budget has to be allocated among multiple competing activities. Since each activity has decreasing marginal returns, we hypothesize that there is an optimum allocation of the budget.

Here, we studied how allocation of a budget to the number of test individuals that are phenotyped and the number of test individuals that are genotyped for performance tests can be optimized in a breeding program for Atlantic salmon. Furthermore, we evaluated the sensitivity of maximum gain in the aggregate genotype and the sensitivity of the relative allocation of budget among activities at the optimum level to (1) the cost of phenotyping in a performance test (2) the cost of genotyping (3) the size of the budget, and (4) the relative emphasis on breeding goal traits. In the "Discussion" section, we elaborate on the mechanisms that underlie optimum allocations strategies and their implications for any breeding program that uses performance tests on sibs of selection candidates.

## Methods

The simulated nucleus breeding program was based on a simplified version of the real-life breeding program of the Norwegian breeding company SalmoBreed, as it was set-up until 2016 (Håvard Bakke, pers. comm. 2018). The breeding program in our analyses used two performance tests: one challenge test for resistance to sea lice (*Lepeophtheirus salmonis*), and one slaughter test. Our aim was to maximize gain in the aggregate genotype ($\Delta\text{H}$) by optimizing allocation of the budget to phenotyping and genotyping in each performance test. First, we describe the optimization and sensitivity analyses, and then we provide a detailed description of the prediction of $\Delta\text{H}$.

### Optimization

$\Delta\text{H}$ is a function of the number of phenotyped full-sibs per family in the challenge test (${n}_{1}$), the number of phenotyped full-sibs per family in the slaughter test (${n}_{2}$), the number of genotyped full-sibs per family in the challenge test (${n}_{3}$), and the number of genotyped full-sibs per family in the slaughter test (${n}_{4}$). Three hundred families were phenotyped each generation. Females were selected based on estimated breeding values (EBV) that were derived from pedigree data only. Males were selected in a two-step procedure: selection was based first on pedigree EBV and second on genomic EBV. Males from only 120 families were selected in the first step, such that the number of families used for genotyping was 120. This two-step selection strategy allows major savings in the costs for genotyping, while it has little effect on $\Delta\text{H}$ [6]. Costs were assumed to be linearly related to the number of records. Costs were €15/fish for ${n}_{1}$, €60/fish for ${n}_{2}$, and €20/fish for genotyping (${n}_{3}$ and ${n}_{4}$). The annual budget for performance tests was €444,000, which was based on the budget necessary to phenotype (300 families) and genotype (120 families) 20 full-sibs per family in the challenge test and 15 full-sibs per family in the slaughter test, as in the SalmoBreed breeding program. Using formal notation, the optimization problem was defined as:

$$\begin{array}{ll} {} & \quad {\text{max}\Delta\text{H}(\mathbf{n})} \\ {\text{subject to}} & \quad {{n}_{i}\in {\mathbb{Z}}_{\ge 0}, \;\;i=1, 2, 3, 4} \\ {} & \quad {300\times \left(15{n}_{1}+60{n}_{2}\right)+120\times 20\times \left({n}_{3}+{n}_{4}\right)\le 444{,}000} \\ {} & \quad {{n}_{1}\ge {n}_{3}} \\ {} & \quad {{n}_{2}\ge {n}_{4}} \end{array}$$

where $\mathbf{n}$ is a vector with elements ${n}_{i}$ ($i=1, 2, 3, 4$), 300 is the number of full-sib families that were phenotyped, and 120 is the number of full-sib families that were genotyped. To solve this optimization problem, we performed a grid search. First, we computed all possible $\mathbf{n}$ at which the budget was exhausted, i.e. when there was no more budget available to increase any of the activities by one unit. This was done by first generating the sequence ${n}_{1,i}$, where $i=0, \dots , {i}_{max}$, with ${i}_{max}$ being the maximum within the budget constraint. For each ${n}_{1,i}$, a sequence ${n}_{2,ij}$ was generated, where $j=0, \dots , {j}_{max}$, with ${j}_{max}$ being the maximum for the remaining budget. For each ${n}_{1,i}$ and ${n}_{2,ij}$, a sequence ${n}_{3,ijk}$ was generated, where $k=0, \dots , {k}_{max}$, with ${k}_{max}$ being the maximum for the remaining budget, provided that it did not exceed ${n}_{1,i}$. Finally, for ${n}_{1,i}$, ${n}_{2,ij}$, and ${n}_{3,ijk}$, a sequence ${n}_{4,ijkl}$ was generated, where $l=0, \dots , {l}_{max}$, with ${l}_{max}$ being the maximum for the remaining budget, provided that it did not exceed ${n}_{2,ij}$. Any **n** for which the budget was not exhausted was removed. We predicted $\Delta\text{H}$ for each remaining $\mathbf{n}$. The point at which $\Delta\text{H}$ reached a maximum returned the optimal solution for $\mathbf{n}$. The maximum $\Delta\text{H}$ and the solutions for $\mathbf{n}$ and ${n}_{i}$ are indicated by an asterisk as: $\Delta{\text{H}}^{{*}}$, ${\mathbf{n}}^{{*}}$, and ${n}_{i}^{*}$.

First, as a simple example, we performed the optimization for pedigree-based selection. In this case, only ${n}_{1}$ and ${n}_{2}$ were allowed to vary, while ${n}_{3}$ and ${n}_{4}$ were set to zero, and $\Delta\text{H}$ was predicted for one-step truncation selection on EBV. Then, we performed the optimization for genomic selection, where ${n}_{1}$, ${n}_{2}$, ${n}_{3}$ and ${n}_{4}$ were allowed to vary. When allocation of the budget was optimized for genomic selection, we estimated the shadow value of the budget constraint, defined as the gain in the objective function $\Delta\text{H}$ from a marginal relaxation of the budget constraint. Because we subsequently found that solutions for ${n}_{3}$ and ${n}_{4}$ were fixed at the boundary, i.e. ${n}_{3}^{*}={n}_{1}^{*}$ and ${n}_{4}^{*}={n}_{2}^{*}$, the shadow value was estimated as the increase in $\Delta\text{H}$ per euro cost of a simultaneous increase by one unit of either ${n}_{1}^{*}$ and ${n}_{3}^{*}$, or ${n}_{2}^{*}$ and ${n}_{4}^{*}$.

In sensitivity analyses, we evaluated the sensitivity of ${\Delta\text{H}}^{{*}}$ and the relative allocation of budget among activities at ${\mathbf{n}}^{*}$ to (1) the accuracy of genomic prediction, by reducing the accuracy (Eq. 1) by 10 or 20%; (2) the cost of phenotyping in the challenge test, by increasing the cost for ${n}_{1}$ from half to double the original cost (€15/fish) in steps of €7.5/fish; (3) the cost of phenotyping in the slaughter test, by increasing the cost for ${n}_{2}$ from half to double the original cost (€60/fish) in steps of €30/fish; (4) the cost of genotyping, by increasing the cost for ${n}_{3}$ and ${n}_{4}$ from half to double the original cost (€20/fish) in steps of €10/fish; (5) the size of the budget, by increasing the budget from zero to double the original budget (€444,000) in steps of €111,000; and (6) the relative emphasis on breeding goal traits, by changing the economic value of sea lice resistance (${\text{R}}_{0})$ by a factor 0.5, 1.5, or 2. In an additional scenario, we used the desired gains [7] to maintain fillet fat content (FilletFat) close to its current level for the index corresponding to $\mathbf{n}{^{\prime}}=[20\,15\,20\,15]$ by deriving the corresponding breeding goal weight for FilletFat by iteration. Then, this weight was used in the breeding goal to find $\Delta{\text{H}}^{{*}}$ in the grid search as before.

To speed up the optimizations, we excluded any $\mathbf{n}$ from the grid search that was unlikely to be ${\mathbf{n}}^{{*}}$, based on previous results. For example, when the original budget was doubled, there were more than 10^5^ possible $\mathbf{n}$ that exhausted the budget, but we expected any new ${n}_{i}^{*}$ to be at least as high as in the solution for the original budget. This reduced the possible $\mathbf{n}$ to only 3017 options, thereby reducing computation time substantially.

### Prediction of gain with the aggregate genotype

The actual SalmoBreed breeding program was more complex than presented in this paper. In this section, we describe only the aspects of the breeding program that were relevant to the prediction of $\Delta\text{H}$ in the context of this study. The breeding goal included thermal growth coefficient (TGC), thermal feed intake coefficient (TFC), fillet yield (FY), and ${\text{R}}_{0}$ for sea lice. TGC is a measure of growth rate that accounts for heterogeneity in smolt weight, rearing period and temperature. TFC is the TGC-analogue for feed intake [8]. FY is the ratio of fillet weight to whole round weight in %. ${\text{R}}_{0}$ is the rate at which sea lice spread across the farmed population, which, combined with the management strategy, determines disease prevalence [9].

Economic values for TGC and TFC were derived using an adapted version of the bio-economic model described in Janssen et al. [8]. The bio-economic model accounted for quota on the number of smolts stocked per cage and on biomass at the farm level. The number of smolts stocked per cage was optimized before and after simulated changes in trait levels. The economic value of FY was derived by substituting $(\text{FY}/100{\%})\times \text{fillet}\;\;\text{price}$ for the price of fish in the profit equation of Janssen et al. [8], such that the economic value equalled the ratio of the sale price of fish over FY. Survival was ignored in the breeding goal, because response in this trait is difficult to predict accurately, while its economic effect was expected to be small [10]. Parameters used to predict $\Delta\text{H}$ are in Table 1.

**Table 1:** Parameters used for each trait to predict the gain in the aggregate genotype

Trait	Baseline trait level (trait unit)	Phenotypic variance (trait unit^2^)	σ_A_ (trait unit)^a^	Economic value (€/trait unit/ton production)	Standardized economic value (€/σ_A_/ton production)
TGC	2.92	0.127	0.208	1780	370
TFC	2.76	0.322	0.272	− 1100	− 299
R_0_	4.7	11.6	1.74	− 65^b^	− 113
FY	69^c^	4.0^d^	1.18	63.8	75.5
DGY	80^c^	2.25^d^	1.10	–	–
FilletFat	17^e^	4.4^e^	1.36	–	–
ViscFat	3.0^e^	0.49^e^	0.336	–	–

*TGC* thermal growth coefficient (g^1/3^/(day degrees × 1000)), *TFC* thermal feed intake coefficient (g^0.317^/(day degrees × 1000)), *R*_*0*_ for sea lice, *FY* fillet yield (%), *DGY* deheaded gutted yield (%), *FilletFat* fillet fat content (%), *ViscFat* visceral fat score (from 0 to 4)

^a^Genetic standard deviation

^b^Janssen et al. [9]

^c^Powell et al. [11]

^d^Haffray et al. [12]

^e^Do [13]

**Fig. 1:**
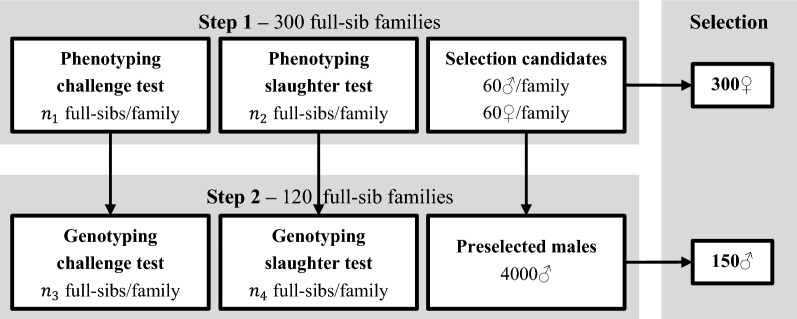
Schematic overview of selection of parents in the nucleus

Year classes in the SalmoBreed breeding program overlapped, resulting in a 3.7 year generation interval. Here, we predicted $\Delta\text{H}$ per year class, assuming that the 4 year classes in the nucleus were discrete. An overview of the annual selection procedure is in Fig. 1. Each year class consisted of 150 males and 300 females. Each male was mated to two females, such that 300 full-sib families were produced. Families were reared in separate tanks until tagging. After tagging, each full-sib family was divided into three groups. From each family, a group of 170 fish was reared as selection candidates, a group of ${n}_{1}$ full-sibs was subjected to a challenge test for sea lice resistance, and a group of ${n}_{2}$ full-sibs was subjected to a slaughter test. All selection candidates had a phenotypic record for TGC. In the challenge test, lice density [14] was recorded to estimate breeding values for ${\text{R}}_{0}$.

In the slaughter test, TGC, deheaded gutted yield (DGY), fillet fat content (FilletFat), and visceral fat score (ViscFat) were recorded. DGY was the ratio of deheaded gutted weight to whole round weight in %, and was used to predict FY. FilletFat and VIscFat were used to predict TFC. From the challenge test, a group of ${n}_{3}$ full-sibs per family was genotyped, and from the slaughter test, a group of ${n}_{4}$ full-sibs per family was genotyped. Genotyping was performed on a 55k SNP array. At the time of selection, 120 candidates per full-sib family had survived and reached sexual maturity. Their sex ratio was 1:1. Females were selected in a single step based on pedigree EBV, obtained using best linear unbiased prediction (BLUP). Males were selected in two steps: selection was first on pedigree EBV using BLUP, and second on genomic EBV using within-family genomic selection, as described in Lillehammer et al. [4]. Genomic selection was applied only in males and not in females, because its impact was strongest on males due their higher selection intensity and because nucleus males were also used to fertilize females in the multiplier tier. Females in the multiplier tier, about 10,000 in total, were mass-selected from about 50,000 female offspring of the very best sires and dams of the nucleus.

Heritabilities and genetic and phenotypic correlations used are in Table 2. Genetic parameters for TGC and TFC were estimated from a simulated bivariate normal distribution for gain in bodyweight and cumulative feed intake at harvest, as in Janssen et al. [10]. The underlying assumptions were a genetic coefficient of variation of bodyweight of 0.15 and a heritability of 0.36 [15]. For the regression of cumulative feed intake at harvest on gain in bodyweight, a slope of 1.17 g feed/g gain, an intercept of 145 g feed, a genetic correlation of 0.70, and phenotypic correlation of 0.60 were assumed. For ${\text{R}}_{0}$, genetic parameters were unknown, but we assumed that a change in the EBV for lice density in the challenge test would give a proportional change in ${\text{R}}_{0}$ [9, 16]. Thus, we used the same phenotypic coefficient of variation and heritability as for lice density [14]. This is equivalent to including lice density in the selection index and ${\text{R}}_{0}$ in the breeding goal, while assuming a genetic correlation of 1 between the two traits.

**Table 2:** Genetic correlations (below diagonal), phenotypic correlations (above diagonal), and heritability estimates (diagonal) for the traits used

	TGC	TFC	${\text{R}}_{0}$	FY	DGY	FilletFat	ViscFat
TGC	0.34	0.56	0	0	0	0.07^a^	0.13^a^
TFC	0.69	0.23	0	0	0	0.06^a^	0.09^a^
${\text{R}}_{0}$	0	0	0.26^b^	0	0	0	0
FY	0	0	0	0.35^c^	0.71^c^	0	0
DGY	0	0	0	0.97^c^	0.54^c^	0	0
FilletFat	− 0.26^a^	0.41^a^	0	0	0	0.42^d^	0.01^d^
ViscFat	0.29^a^	0.09^a^	0	0	0	0.01^d^	0.23^d^

*TGC* thermal growth coefficient (g^1/3^/(day degrees × 1000)), *TFC* thermal feed intake coefficient (g^0.317^/(day degrees × 1000)), ${{R}}_{0}$ for sea lice, *FY* fillet yield (%), *DGY* deheaded gutted yield (%), *FilletFat* fillet fat content (%), *ViscFat* visceral fat score (from 0 to 4)

^a^Kause et al. [17]

^b^Gjerde et al. [14]

^c^Haffray et al. [12]

^d^Do [13]

Breeding programs that apply genomic selection can be simulated deterministically by using extended selection index theory, as outlined by Dekkers [18]. For each trait for which phenotypes were recorded (TGC, ${\text{R}}_{0}$, DGY, FilletFat, and ViscFat), an additional genomic trait was added to the selection index. Each of these genomic traits had a heritability of 0.999, a phenotypic variance of 1, and a genetic correlation with the corresponding breeding goal trait equal to the expected accuracy of genomic prediction for that trait (${r}_{GS}$). Hayes et al. [19] developed deterministic equations to estimate ${r}_{GS}$ from full- and half-sib records, but here we ignored the information from half-sib records, because they were not available for some of the preselected males. The genomic prediction accuracy for $m$ full-sib records was estimated as in [19], using:

1 $${r}_{GS}=\sqrt{\frac{0.5\times m}{m-1+2/{h}^{2}}+\frac{0.5\times m}{m+4\times {M}_{e}/{h}^{2}}}$$

where ${M}_{e}$ is the effective number of chromosome segments that segregate independently in the population, which was estimated from the variation in kinship among full-sibs ($V\left({P}_{FS}\right)$) as in Hayes et al. [19]:

2 $${M}_{e}=\frac{1}{8\times V\left({P}_{FS}\right)}$$

where $V\left({P}_{FS}\right)$ was estimated as in Hill [20]:

3 $$V\left({P}_{FS}\right)=\frac{4\times L-{N}_{chr.}+\sum_{i=1}^{{N}_{chr.}}{e}^{-4\times {l}_{i}}}{64\times {L}^{2}}$$

where $L$ is the total length of the genome in Morgan (M), ${N}_{chr.}$ is the number of chromosomes, and ${l}_{i}$ is the length of chromosome $i$. For Atlantic salmon, $L=3.9$ M in males and $L=19.83$ M in females, ${N}_{chr.}$ is equal to 29, and values for ${l}_{i}$ were obtained from Moen et al. [21]. ${M}_{e}$ was calculated separately for each sex and averaged, resulting in ${M}_{e}=37.5$.

To compute $${r}_{GS}$$ for TGC, we made the simplifying assumption that the number of preselected male selection candidates was the same for each of the 120 remaining families, such that the number of genotyped selection candidates per family was 33, i.e. 32 full-sibs per candidate. An additional $${n}_{4}$$ full-sibs per family from the slaughter test had records for TGC, hence $${r}_{GS}$$ for TGC was computed for $$32+{n}_{4}$$ full-sibs per family. For $${\text{R}}_{0}$$, $${r}_{GS}$$ was based on records for $${n}_{3}$$ full-sibs per family. For DGY, FilletFat, and ViscFat, $${r}_{GS}$$ was based on records for $${n}_{4}$$ full-sibs per family. Correlations of genomic traits with other traits were derived using path coefficients [18]. A table with all the correlations for the optimum index for genomic selection is in Appendix. As an example, for DGY with $${n}_{4}=16$$$${r}_{GS}=\sqrt{\frac{0.5\times 16}{16-1+2/{0.54}^{2}}+\frac{0.5\times 16}{16+4\times 37.5/{0.54}^{2}}}=0.674$$ (Eq. 1).

Thus, the corresponding index included a genomic trait with a heritability of 0.999, a phenotypic variance of 1, a genetic correlation with DGY of 0.674, a phenotypic correlation with DGY of $0.674\times \sqrt{0.54}=0.50$, a genetic correlation with FY of $0.674\times 0.97=0.65$, and a phenotypic correlation with FY of $0.65\times \sqrt{0.35}=0.39$.

Gain in the aggregate genotype, $\Delta\text{H}$, was predicted by deterministic simulation of two-step selection in SelAction [22]. Selected proportions ($p$) for males were set to $p=\frac{4000}{{18,000}}=0.22$ in the first step and to $p=\frac{150}{4000}=0.038$ in the second step, and for females $p=0.999$ in the first step and $p=\frac{300}{{18,000}}=0.017$ in the second step. For all non-genomic traits, the proportion of phenotypic variance explained by the effects that were common to full-sibs (${\text{c}}^{2})$ was set to 0.05. Selection indices are in Table 3. The rate of inbreeding was not evaluated, because it is not computed by SelAction for two-step selection and, in reality, generations overlapped.

**Table 3:** Selection indices used in the nucleus breeding program

Trait	Records	Females and 1st step males	2nd step males
TGC	Own performance	✔	✔
	Pedigree index^a^	✔	✔
	119 full-sibs	✔	✔
	120 half-sib	✔	✔
	GEBV^b^ for 32 + n_4_ full-sibs		✔
${\text{R}}_{0}$	Pedigree index	✔	✔
	${n}_{1}$ full-sibs	✔	✔
	${n}_{1}$ half-sibs	✔	✔
	GEBV for ${n}_{3}$ full-sibs		✔
TGC, DGY, FilletFat, and ViscFat	Pedigree index	✔	✔
	${n}_{2}$ full-sibs	✔	✔
	${n}_{2}$ half-sibs	✔	✔
	GEBV for ${n}_{4}$ full-sibs		✔

*TGC* thermal growth coefficient (g^1/3^/(day degrees × 1000)), ${{R}}_{0}$ for sea lice, *DGY* deheaded gutted yield (%), *FilletFat* fillet fat content (%), *ViscFat* visceral fat score (from 0 to 4)

^a^SelAction condenses all ancestral information into estimated breeding values of parents as described by Wray and Hill [23]. In SelAction, this is termed ‘BLUP’.

^b^Breeding value estimated using within-family genomic prediction

## Results

### Pedigree-based selection

When the objective was to maximize $\Delta\text{H}$ with pedigree-based selection (Table 4), the optimum number of phenotyped full-sibs per family was equal to 22 in the challenge test (${n}_{1}^{*}$) and 19 in the slaughter test (${n}_{2}^{*}$). $\Delta{\text{H}}^{{*}}$ was equal to €351/ton, corresponding to €95/ton per year for a 3.7 year generation interval, of which 81% was due to improvement of TGC and TFC. The combined effect of gains in TGC and TFC reduced feed conversion ratio (FCR) from 1.20 to 1.08 (results from the bio-economic model). The gain in ${\text{R}}_{0}$ corresponds to an expected reduction in treatment frequency from 3.7 to 3.3 treatments per production cycle, while the average number of lice remains unchanged.

**Table 4:** Genetic gain per generation in the optimized breeding program for Atlantic salmon using pedigree or genomic selection

Trait	Pedigree-based selection		Genomic selection	
	Genetic gain (σ_A_)	Genetic gain (€/ton production)	Genetic gain (σ_A_)	Genetic gain (€/ton production)
TGC	0.90	331.6	0.96	355.8
TFC	0.16	− 47.0	0.15	− 43.7
${\text{R}}_{0}$	− 0.39	44.3	− 0.44	49.5
FY	0.29	21.7	0.32	24.2
DGY	0.30		0.33	
FilletFat	− 0.92		− 1.02	
ViscFat	0.35		0.39	
Total		351		386

*TGC* thermal growth coefficient (g^1/3^/(day degrees × 1000)), *TFC* thermal feed intake coefficient (g^0.317^/(day degrees × 1000)), ${{R}}_{0}$ for sea lice, *FY* fillet yield (%), *DGY* deheaded gutted yield (%), *FilletFat* fillet fat content (%), *ViscFat* visceral fat score (from 0 to 4)

**Fig. 2:**
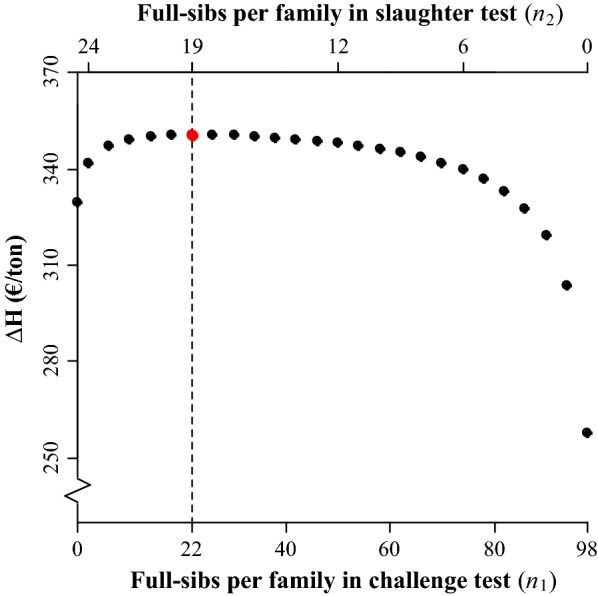
Gain in the aggregate genotype ($\Delta\text{H}$) for varying numbers of phenotyped full-sibs per family used in performance tests. The vertical dashed line indicates the optimum and the red point is at $\Delta{\text{H}}^{{*}}$

The $\Delta\text{H}$ for any combination of ${n}_{1}$ and ${n}_{2}$ that exhausted the (fixed) budget is in Fig. 2. At the maximum value of $\Delta\text{H}$, most of the budget was allocated to the slaughter test. Around the maximum value of $\Delta\text{H}$, the curve in Fig. 2 is relatively flat, which means that $\Delta\text{H}$ was rather robust to changes in budget allocation to ${n}_{1}$ and ${n}_{2}$. However, at both extremes, and particularly with few full-sibs per family in the slaughter test, $\Delta\text{H}$ was compromised. Marginal gains from phenotyping an extra full-sib per family were relatively high when the number of full-sibs per family was small, and decreased as it increased. The selection intensity was constant for any combination of ${n}_{1}$ and ${n}_{2}$ and, therefore, differences in $\Delta\text{H}$ were largely due to differences in the accuracy of the index and to a minor extent due to differences in genetic variation in the breeding goal caused by the Bulmer-effect (results from SelAction output).

### Genomic selection

When the objective was to maximize $\Delta\text{H}$ with genomic selection (Table 4), the optimum number of phenotyped and genotyped full-sibs per family was equal to 17 in the challenge test (${n}_{1}^{*}$ and ${n}_{3}^{*}$) and 16 in the slaughter test (${n}_{2}^{*}$ and ${n}_{4}^{*}$), so that ${\mathbf{n}}^{{*}}{^{\prime}}=[17\text{ }16\text{ }17\text{ }16]$. $\Delta{\text{H}}^{*}$ was €386/ton, corresponding to €104/ton per year for a 3.7 year generation interval, which represents an increase of 10% compared to pedigree-based selection, mostly due to extra gain in TGC. The shadow value of the budget constraint was €3.1 × 10^–5^/ton per euro costs when estimated from a one unit increase in ${n}_{1}^{*}$ and ${n}_{3}^{*}$, and €3.4 × 10^–5^/ton per euro costs when estimated from a one unit increase in ${n}_{2}^{*}$ and ${n}_{4}^{*}$.

**Fig. 3:**
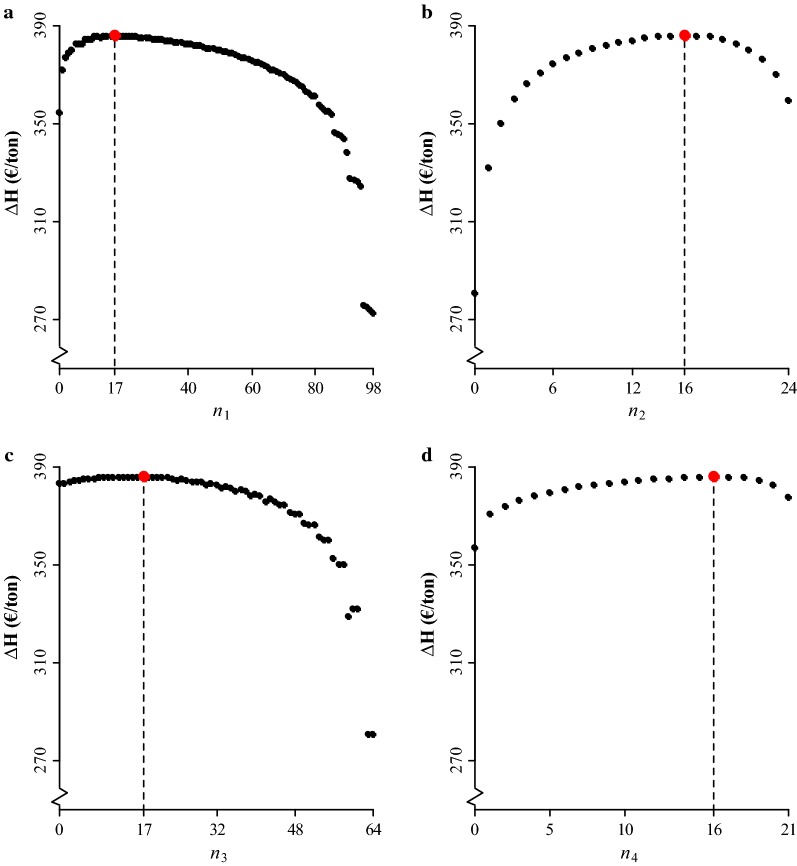
Maximum gain in the aggregate genotype ($\Delta\text{H}$) for varying numbers of **a** phenotyped full-sibs per family in the challenge test (${n}_{1}$), **b** phenotyped full-sibs per family in the slaughter test (${n}_{2}$), **c** genotyped full-sibs per family in the challenge test (${n}_{3}$), **d** genotyped full-sibs per family in the slaughter test (${n}_{4}$). The vertical dashed lines and red points indicate the optimum

Figure 3 gives the maximum $\Delta\text{H}$ for varying numbers of full-sibs per family for each of the four activities. At all maxima, the number of full-sibs per family in the other three activities was optimum for the given value of the activity. As in the case of pedigree-based selection, the curve is relatively flat around the maximum value of $\Delta\text{H}$, which means that $\Delta\text{H}$ was rather robust to changes in budget allocation among $\mathbf{n}$ around the optimum.

### Sensitivity analyses

**Fig. 4:**
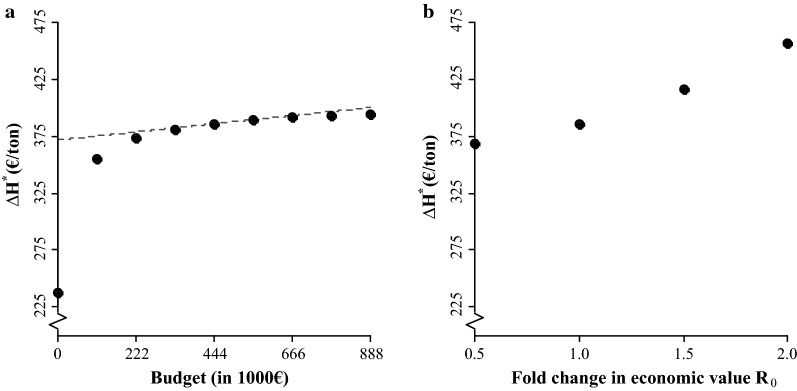
Sensitivity of gain in the aggregate genotype at the optimum (${\Delta\text{H}}^{{*}}$) to **a** the budget, where the slope of the dashed line is equal to the shadow value of the budget constraint, **b** the economic value of ${\text{R}}_{0}$

**Fig. 5:**
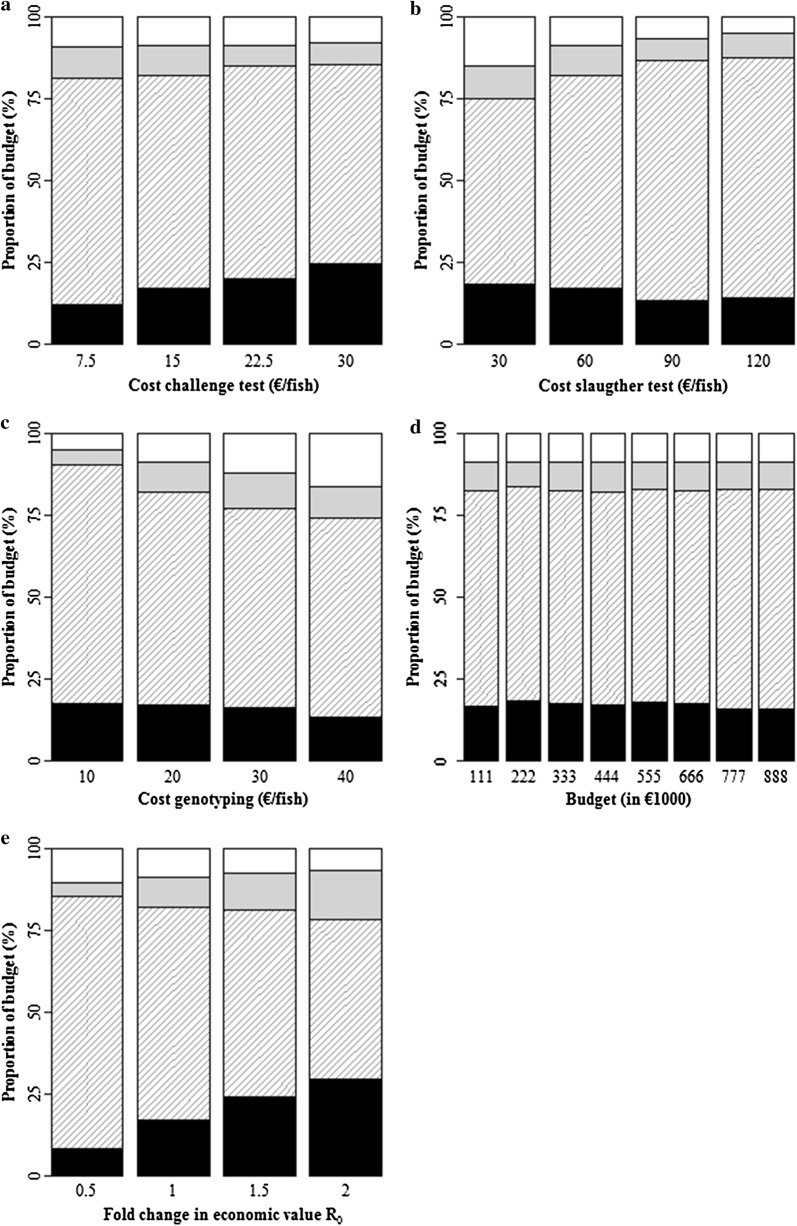
The proportion of budget allocated to activities (${n}_{1}^{*}$ = black,  ${n}_{2}^{*}$ = grey stripes,  ${n}_{3}^{*}$ = grey, ${n}_{4}^{*}$ =  white) when the allocation of budget has been optimized **a** for increasing costs of phenotyping in the challenge test, **b** for increasing costs of phenotyping in the slaughter test, **c** for increasing costs of genotyping, **d** for an increasing size of the budget, **e** for an increasing economic value of ${\text{R}}_{0}$

When the accuracy of genomic prediction decreased by 20%, $\Delta{\text{H}}^{{*}}$ decreased by 3.3% (Table 5). $\Delta{\text{H}}^{{*}}$ was rather insensitive to the cost of phenotyping in the challenge test, the cost of phenotyping in the slaughter test, and the cost of genotyping, i.e. it decreased only marginally as these costs increased. When the budget for the performance tests was zero, i.e. there were no performance tests, $\Delta\text{H}$ was €237/ton, corresponding to 62% of $\Delta{\text{H}}^{{*}}$ at a budget of €444,000 (Fig. 4a). $\Delta{\text{H}}^{{*}}$ increased when the size of the budget increased but at a declining rate. The slope of the relationship between ${\Delta\text{H}}^{{*}}$ and size of the budget, at a budget of €444,000, equalled the average shadow value of the budget constraint. Furthermore, $\Delta{\text{H}}^{{*}}$ itself became more sensitive to changes in budget allocation among $\mathbf{n}$ around the optimum when the budget decreased, and more robust when the budget increased. When the economic value of ${\text{R}}_{0}$ increased and allocation of the budget among activities remained constant, $\Delta\text{H}$ increased at an increasing rate. This trend was somewhat more pronounced for $\Delta{\text{H}}^{{*}}$ (Fig. 4b), because an increasing proportion of the budget was allocated to the challenge test (Fig. 5f). Note that if the actual economic value would remain constant while only the emphasis on ${\text{R}}_{0}$ is increased, $\Delta{\text{H}}^{{*}}$ would decrease instead.

**Table 5:** Effect of parameters tested in the sensitivity analyses on ${{\Delta}}{\text{H}}^{{*}}$ and ${\text{n}}^{{*}}{^{\prime}}$

Item	Change	${{\Delta}}{\text{H}}^{{*}}$ (€/ton)	${\text{n}}^{{*}}{^{\prime}}$
Base	None	386	[17 16 17 16]
Accuracy genomic prediction (Eq. 1)	− 10%	379	[17 16 17 16]
	− 20%	373	[17 16 17 16]
Cost of challenge test sea lice	− 50%	387	[24 17 18 17]
	+ 50%	385	[13 16 12 16]
	+ 100%	384	[12 15 12 15]
Cost of slaughter test	− 50%	392	[18 28 18 28]
	+ 50%	381	[13 12 13 12]
	+ 100%	377	[14 9 14 9]
Cost of genotyping	− 50%	387	[17 18 17 18]
	+ 50%	384	[16 15 13 15]
	+ 100%	383	[13 15 9 15]

The relative allocation of the budget among activities at ${\mathbf{n}}^{*}$ was unaffected by a 20% reduction in the accuracy of genomic prediction (Table 5). Figure 5 shows the relationship of the relative allocation of the budget among activities at ${\mathbf{n}}^{*}$ with the other parameters evaluated in the sensitivity analysis. When the cost of phenotyping in the challenge test increased, an increasing proportion of the budget was spent on ${n}_{1}^{{*}}$ (Fig. 5a). When the cost of phenotyping in the slaughter test increased, an increasing proportion of the budget was spent on ${n}_{2}^{{*}}$ (Fig. 5b). When the cost of genotyping increased, an increasing proportion of the budget was spent on ${n}_{3}^{{*}}$ and ${n}_{4}^{{*}}$ (Fig. 5c). For a fourfold change in costs of genotyping, the reduction in ${n}_{3}^{*}$ and ${n}_{4}^{*}$ was relatively small (Table 5). When the size of the budget increased, the relative allocation of the budget among activities remained fairly constant (Fig. 5d). When the economic value of ${\text{R}}_{0}$ increased, the proportion of budget spent on the challenge test (${n}_{1}^{*}$ and ${n}_{3}^{*}$) increased, at the expense of the slaughter test (${n}_{2}^{*}$ and ${n}_{4}^{*}$) (Fig. 5e).

For the desired gains index, the weight given to FilletFat was €170/%/ton production, which resulted in a negligible change in FilletFat, but TFC increased more than in the baseline where FilletFat had zero weight in the breeding goal. As a result, $\Delta{\text{H}}^{*}$ was €242/ton for the desired gains index, which represented a decrease of 37% relative to the baseline. For the desired gains index, relative contributions of TGC and TFC to $\Delta{\text{H}}^{{*}}$ decreased to 50% compared to 81% in the baseline, whereas relative contributions of ${\text{R}}_{0}$ and FY increased. At the maximum $\Delta\text{H}$ for the desired gains index, more budget was allocated to the challenge test, at the expense of the slaughter test, such that ${\mathbf{n}}^{{*}}{^{\prime}}=[29\;12\;28\;12]$.

## Discussion

Results for optimization of the breeding program showed that there is an optimum allocation of the budget to phenotyping and genotyping effort in performance tests that maximizes gain in the aggregate genotype. The curves of $\Delta$H were rather flat around the optima for the scenarios evaluated, hence gains in the aggregate genotype were relatively robust to changes in budget allocation among activities around the optimum value. However, at the extremes, i.e. when the number of full-sibs per family in one of the activities was very small or very large, gains were substantially lower. Although potential gains from optimizing phenotyping and genotyping efforts for a fixed budget may be small, they come at no extra cost (except for the optimization itself). Sensitivity analyses showed that maximum gain in the aggregate genotype was sensitive to the size of the budget (Fig. 4a) and to the relative emphasis on breeding goal traits (Fig. 4b), but was less sensitive to the accuracy of genomic prediction and the costs of phenotyping and genotyping (Table 5). The relative allocation of budget among activities at the optimum was sensitive to the costs of phenotyping and genotyping (Fig. 5a–c) and the relative emphasis on breeding goal traits (Fig. 5e), but was less sensitive to the accuracy of genomic prediction (Table 5) and the size of the budget (Fig. 5d). At the optimum, the numbers of phenotyped and genotyped full-sibs were similar for both performance tests, but due to different costs of phenotyping, most of the budget was allocated to the slaughter test. For both performance tests, the optimum number of full-sibs that was genotyped was equal to the number that was phenotyped.

The results of this study can be better understood when considering the underlying mechanism of the four activities. Accuracy of selection increases at a declining rate with number of individuals allocated to each of the four activities. Any increase in accuracy increases gain in the aggregate genotype. Thus, gain in the aggregate genotype is maximum at some point where the budget is exhausted. When the budget is exhausted, a further increase in one of the activities should coincide with a decrease in other activities to meet the budget constraint. At the optimum, marginal gains in the aggregate genotype per unit cost are approximately equal for all activities that are bounded by the same constraints, and any deviation thereof is due to the non-continuous nature of the objective function. Since for both performance tests marginal gains per unit cost of genotyping were higher than those per unit cost of phenotyping but the number of genotyped full-sibs was bounded by the number of phenotyped full-sibs, the optimum number of genotyped full-sibs per family equalled the optimum number of phenotyped full-sibs per family. With equal numbers of phenotyped and genotyped full-sibs per family per performance test (${n}_{1}={n}_{3}$ and ${n}_{2}={n}_{4}$), gain in the aggregate genotype is a function of only two variables, which are both only bounded by the budget constraint. At the optimum, marginal gains per unit costs of these two variables are approximately equal, as evidenced by the similar shadow values calculated from a simultaneous increase by one unit of either ${n}_{1}^{*}$ and ${n}_{3}^{*}$ (€3.1 × 10^–5^/ton), or ${n}_{2}^{*}$ and ${n}_{4}^{*}$ (€3.4 × 10^–5^/ton). When marginal gains per unit costs are equal, moving budget from one activity to another has no effect on gain in the aggregate genotype, i.e. what is lost by decreasing the budget for one activity is gained by increasing the budget for the other activity. Since marginal gains per unit cost converge for all activities (bounded by the same constraints) when approaching the optimum, moving budget from one activity to the other has little effect on gain in the aggregate genotype around the optimum. Thus, when costs of phenotyping or genotyping change, budget can be reallocated among activities (Fig. 5a–c), such that maximum gain in the aggregate genotype does not change much (Table 5). However, when costs of all activities change simultaneously in the same direction, which is equivalent to a change in size of the budget, the maximum gain in the aggregate genotype will change (Fig. 4a). The above mechanism explains why the curve for $\Delta$H is rather flat around the optimum (Figs. 2 and 3). This result is consistent with the few studies in the literature that focused on the economic optimization of test group sizes. Some of the older studies that dealt with optimization of cattle and pig breeding programs were reviewed by Cunningham [24] and Lindhé and Holmquist-Arbrandt [25], respectively. More recently, De Vries and Van Der Steen [26] evaluated the distribution of testing capacity across a sire and dam line in pig breeding for a fixed total testing capacity. In their optimizations, they did not compute the full range of possible solutions, hence their solutions may have been somewhat off the optimum. However, differences in the objective function (the selection response) between scenarios were small close to the optimum. Dekkers et al. [2] optimized the size of progeny groups in a dairy cattle breeding program and computed several points on the response surface, which they interpolated. For a large range of options, such an approach may be more efficient than the grid search in our study. However, we managed to keep the feasible region of the solution relatively small by using previous results, such that the grid search was not so computation intensive. Similar to our study, De Vries and Van Der Steen [26] and Dekkers et al. [2] found that marginal gains in the objective function decreased with increasing group size, and that the curve of the objective function was relatively flat around the optimum.

The simulated breeding program used within-family genomic selection, for which the reference population was limited to sibs of selection candidates. Alternatively, a breeding program could use population-wide genomic selection, for which the reference population would include both sibs of selection candidates and largely unrelated individuals. In population-wide genomic selection, the reference population increases in size when additional animals are genotyped. The value of these additional animals depends strongly on their relatedness to selection candidates [27], which decreases over time, such that older generations contribute relatively little to the overall accuracy. Furthermore, the relatedness between individuals in the reference population and selection candidates is higher within than between year classes. Thus, sibs of selection candidates contribute most to the accuracy of genomic prediction. This is evidenced by similar accuracies of population-wide and within-family genomic prediction in salmon breeding programs [28]. The accuracy of population-wide genomic prediction is difficult to estimate deterministically, whereas the accuracy of within-family genomic prediction can be estimated more precisely, as in our study (Eq. 1, [19]). Since accuracies of population-wide and within-family genomic prediction are similar but deterministic methods are preferable for optimization, within-family genomic prediction may be assumed here, even when population-wide genomic prediction is used in practice.

To predict genetic gain in ${\text{R}}_{0}$ for sea lice, some assumptions were inevitable. We assumed that lice density is genetically the same trait as susceptibility in field conditions, which is supported by a genetic correlation of 0.88 between lice count in a challenge test and under field conditions [16]. We assumed that susceptibility and infectivity were genetically uncorrelated, although these traits might have the same genetic basis [9]. Susceptibility and infectivity have multiplicative effects on ${\text{R}}_{0}$, such that a genetic correlation of 1 between susceptibility and infectivity approximately doubles genetic progress in ${\text{R}}_{0}$ [29]. For example, with a genetic correlation of 1, a 10% improvement in lice density reduces both susceptibility and infectivity by 10%, such that ${\text{R}}_{0}$ reduces by $\left(1-{0.90}^{2}\right)\times 100\%=19\%$. The economic effect of a genetic correlation of 1 can thus be predicted by doubling the economic value of ${\text{R}}_{0}$, which is covered by the conducted sensitivity analysis. If the genetic correlation between susceptibility and infectivity is equal to 1, more budget needs to be allocated to the challenge test (Fig. 5e) and $\Delta{\text{H}}^{*}$ increases to €456/ton (Fig. 4b).

The objective of the optimization was to maximize gain in the aggregate genotype averaged over males and females, which maximizes the rate of genetic gain in the nucleus. Since nucleus males are also used in the multiplier tier, the strategy of genotyping males shortens the genetic lag between the nucleus and multiplier tier. Furthermore, it allows the breeding company to create production lines that are selected on quantitative trait loci for specific disease resistance. However, for the above objective, it would be relevant to test how much extra gain could be achieved by genotyping also female nucleus selection candidates. Similar to the selection of males, two-step selection could be used for females, with the second step based on within-family genomic selection. Such two-step selection would increase the genetic selection differential for females relative to pedigree-based selection, although somewhat less than for males due to the lower selection intensity in females. When preselected females are taken from the same full-sib families as preselected males, the increase in costs could be limited to the costs of genotyping preselected females, while making better use of genotyped full-sibs in performance tests. Such a strategy would, however, increase the rate of inbreeding, because the first selection step would force preselected females to belong to the same families as preselected males. The rate of inbreeding, however, can be controlled by other measures, such as optimum contribution selection [30]. Thus, genotyping a preselected fraction of female selection candidates may be a cost-effective option to further accelerate genetic gain in the nucleus.

When FilletFat was given zero weight in the breeding goal, records of fat content in the slaughter test served to explain variation in TFC that was not explained by variation in TGC. The consequence was that FilletFat decreased (Table 4) and FCR improved, which agrees with theory [31]. In the desired gains index, the positive genetic correlation between FilletFat and TFC was antagonistic, because weights on FilletFat and TFC had opposite signs, whereas this correlation was not antagonistic when FilletFat had zero weight. Therefore, the proportion of variation in the breeding goal explained by a given number of phenotyped or genotyped individuals in the slaughter test was lower in the desired gains index than when FilletFat had zero weight. As a result, the relative importance of the slaughter test decreased in the desired gains index and at the optimum, a larger proportion of the budget was allocated to the challenge test, at the expense of the slaughter test.

The optimization of full-sib test group size and genotyping effort is not restricted to breeding objectives based on economic values but can also be used for any desired gains index. In the desired gains index used here, the weight given to FilletFat may be perceived as its shadow price [32]. This shadow price indicates the maximum amount that producers can afford to pay for a marginal change in FilletFat, i.e. the value of a 1% increase with other traits held constant. In other words, the weight given to FilletFat implies that producers would expect the sale price of fish to decrease by €170/ton when FilletFat decreases by 1%. Underlying this shadow price is the assumption of a negative genetic correlation between TGC and FilletFat. This genetic correlation is negative when genetic improvement of TGC leads to a shorter time interval to reach a constant harvest weight, but it is positive when, instead, genetic improvement of TGC leads to a greater harvest weight in a constant growing period [17, 33, 34]. If a positive genetic correlation was more appropriate, FilletFat would increase when its economic value is zero and a negative weight would be required to keep its level constant. Thus, the shadow price of FilletFat strongly depends on the genetic correlation of FilletFat with TGC, which depends on an uncertain management response to improvement of TGC.

Based on our predictions, the transition from pedigree-based to genomic selection increased the rate of genetic gain by 10% per generation. This increase is only moderate, because TGC contributed most to gain in the aggregate genotype and its accuracy was already high with pedigree-based selection. Still, most extra gains from genomic selection were due to extra gain in TGC. Thus, although genomic selection may be particularly useful for traits that cannot be recorded on selection candidates, most of the extra gain may come from a slightly higher accuracy on a trait that already dominates gain in the aggregate genotype in pedigree-based selection, such as TGC in this study. For the optimized genomic selection program, gain in the aggregate genotype was €386/ton per generation. This is an optimistic estimate of the increase in benefits from genetic improvement, because it was computed as if economic values were used to balance the emphasis on breeding goal traits in an optimal way, which may not be the case in practice. In practice, for example, FilletFat is held constant using desired gains. For the optimized desired gains index that keeps FilletFat constant, gain in the aggregate genotype was €242/ton per generation. For a generation interval of 3.7 years, as in the SalmoBreed breeding program, this would correspond to an increase in benefits of about €66/ton per year. This estimate is only slightly higher than a previous estimate of €50/ton per year reported by Gjerde et al. [35], which was a crude estimate based on realized gains in growth and feed conversion ratio. If we assume a yield of 3.8 kg product per egg, the genetic value of an egg increases by about €0.25 per year. In contrast, the sale price of eggs is about €0.18/egg and has increased by only ~ €0.01 per year over the last couple of years. This suggests that only a minor proportion of the benefits of genetic improvement are accrued by the breeding company, while most of the benefits are passed on to fish producers and consumers. In the short term, benefits from genetic improvement may be accrued by fish producers, when genetic improvement generates a competitive advantage. These benefits may be passed on to the consumer in the long term when competition pushes profit margins downwards [36]. The uneven distribution of benefits from genetic improvement between the breeding company and the fish producers causes underinvestment in genetic improvement. If the breeding company received a premium of €0.01/egg for its annual sales of 120 million eggs, it would accrue an extra 1.2 million euro. The shadow value of the budget constraint on performance tests was about €3.2 × 10^–5^/ton per euro costs. This means that if gain in the aggregate genotype increased linearly with size of the budget, it would increase by €3.2/ton per €100,000 costs. Thus, assuming linearity, with a premium of €0.01/egg, the use of the extra returns to increase the budget for performance tests could increase gain in the aggregate genotype by €1.2 million × €3.2 × 10^–5^/ton per euro costs, which equals €38/ton per generation. Using the same assumptions as before, a premium of €0.01/egg would increase the genetic potential of an egg by an extra (€0.038/kg per generation × 3.8 kg/egg)/(3.7 year per generation), which equals €0.039/egg per year. Such higher genetic gains would improve the competitive position of the breeding company, while at the same time increasing the benefits to producers. As long as discounted benefits from extra genetic gain exceed the discounted costs, such a premium on genetic superiority would be profitable for both the breeding company and producers.

## Conclusions

An optimum allocation of a fixed budget to phenotyping and genotyping efforts in performance tests exists that maximizes gain in the aggregate genotype. Maximum gain in the aggregate genotype was sensitive to the size of the budget and to the relative emphasis on breeding goal traits, but was less sensitive to the accuracy of genomic prediction and the costs of phenotyping and genotyping. The relative allocation of budget among activities at the optimum was sensitive to the cost of phenotyping and genotyping and to the relative emphasis on breeding goal traits, but was less sensitive to the accuracy of genomic prediction and the size of the budget. Although potential gains from optimizing phenotyping and genotyping efforts may be small, under a fixed budget, they come at no extra cost.

## Data Availability

All data generated or analysed during this study are included in this published article.

## Appendix

See Table 6.

**Table 6:** Genetic correlations (below diagonal), phenotypic correlations (above diagonal), and heritabilities (diagonal) of traits and their genomic selection counterparts for n*′ = [16 17 16 17]

	TGC	TFC	${\text{R}}_{0}$	FY	DGY	FilletFat	ViscFat	G_TGC	$\text{G}\_{\text{R}}_{0}$	G_DGY	G_FilletFat	G_ViscFat
TGC	0.34	0.56	0	0	0	0.07	0.13	0.41	0	0	− 0.10	0.10
TFC	0.69	0.23	0	0	0	0.06	0.09	0.23	0	0	0.13	0.03
${\text{R}}_{0}$	0	0	0.26	0	0	0	0	0	0.31	0	0	0
FY	0	0	0	0.35	0.71	0	0	0	0	0.39	0	0
DGY	0	0	0	0.97	0.54	0	0	0	0	0.50	0	0
FilletFat	− 0.26	0.41	0	0	0	0.42	0.01	− 0.12	0	0	0.42	0
ViscFat	0.29	0.09	0	0	0	0.01	0.23	0.10	0	0	0	0.28
G_TGC	0.71	0.49	0	0	0	− 0.18	0.21	0.999	0	0	− 0.12	0.12
$\text{G}\_{\text{R}}_{0}$	0	0	0.61	0	0	0	0	0	0.999	0	0	0
G_DGY	0	0	0	0.65	0.67	0	0	0	0	0.999	0	0
G_FilletFat	− 0.17	0.27	0	0	0	0.65	0.01	− 0.12	0	0	0.999	0
G_ViscFat	0.17	0.05	0	0	0	0.01	0.59	0.12	0	0	0.00	0.999

*TGC* thermal growth coefficient (g^1/3^/(day degrees × 1000)), *TFC* thermal feed intake coefficient (g^0.317^/(day degrees × 1000)), ${{R}}_{0}$ for sea lice, *FY* fillet yield (%), *DGY* deheaded gutted yield (%), *FilletFat* fillet fat content (%), *ViscFat* visceral fat score (from 0 to 4), *G_trait* trait for which within-family genomic prediction was used to estimate breeding values
